# Clinical potential of pupillary light reflex parameters as objective indicators reflecting chronic rhinosinusitis-specific quality of life: a 12-month prospective longitudinal study

**DOI:** 10.1038/s41598-021-01153-1

**Published:** 2021-11-03

**Authors:** Hiroatsu Hatsukawa, Masaaki Ishikawa

**Affiliations:** Department of Otolaryngology, Head and Neck Surgery, Hyogo Prefectural Amagasaki General Medical Center, Higashinaniwachou, 2-17-77, Amagasaki, Hyogo 6608550 Japan

**Keywords:** Medical research, Neurology

## Abstract

Pupillary light reflex (PLR) and heart rate variability (HRV) parameters can be objective indicators of chronic rhinosinusitis (CRS) status from the viewpoint of autonomic nervous system activity. This study aimed to establish objective indicators for CRS using the 22-item Sino-Nasal Outcome Test (SNOT-22) and PLR/HRV parameters. Sixty-seven patients were prospectively and longitudinally followed up after surgical treatment. We investigated changes in SNOT-22 scores, representing CRS-specific quality of life (QOL). We prepared two models: linear regression model adjusting clinical factors as predictor variables (model 1) and linear mixed-effects model adjusting clinical factors and among-individual variability (model 2). We compared Akaike’s information criterion (AIC) values and regression coefficients. The model with lower AIC values was defined as the better-fit model. Model 2 showed lower AIC values in all parameters (better-fit model). Three parameters showed opposite results between the two models. The better-fit models showed significances in the five PLR parameters but not in any HRV parameters. Among these PLR parameters, constriction latency can be the most robust indicator because of the narrowest 95% confidence intervals. Adjusting the among-individual variability while investigating clinical potential of PLR/HRV parameters to reflect CRS-specific QOL can improve the model fit, thereby reaching robust conclusions from obtained data.

## Introduction

The nose possesses rich autonomic innervation of the nasal vasculature and nasal glands, contributing to the regulation of temperature and humidification of the air entering the lower airways^1–3^. Therefore, dysfunction of autonomic nervous system (ANS) activity may be associated with nasal symptoms and disorders. Chronic rhinosinusitis (CRS) is a representative upper-airway disorder characterized by a persisting inflammatory condition of the paranasal sinus. Some studies have investigated the association between CRS and ANS activity using tissue samples^4,5^ and subjective tools^6^.

One clinical study investigated the association between ANS dysfunction and CRS using the 31-item Composite Autonomic Symptom Score (COMPASS-31), a validated questionnaire for evaluating ANS dysfunction^6^. The results showed significant correlations between the 22-item Sino-Nasal Outcome Test (SNOT-22) and COMPASS-31 in participants with CRS with nasal polyps. Thus, these questionnaires can be used as subjective tools for assessing CRS-related ANS dysfunction, although they may be subject to bias. Therefore, the establishment of objective indicators reflecting CRS-related ANS activity can be helpful to obtain novel insights into the association between CRS and ANS activity. As the cardiac status and pupillary status are controlled by ANS activity^7–10^, recent studies investigated the association between ANS dysfunction and upper-airway disorders, such as obstructive sleep apnoea syndrome^11–13^ and allergic rhinitis^14,15^, using autonomic function tests for cardiac and/or pupillary status. However, to date, no clinical study has explored the association between ANS activity and CRS using objective indicators.

The pupillary light reflex (PLR) and/or heart rate variability (HRV) can be effective measures in investigating the association between ANS activity and physiological burden, such as that resulting from exercise^16^, foot bath^17^, and pain^18^. PLR is controlled by two kinds of muscles via the locus coeruleus containing both excitatory sympathetic premotor neurons and inhibitory parasympathetic premotor neurons, which is located in the brainstem: the iris sphincter muscle innervated by the parasympathetic nervous system (PNS) and the iris dilator muscle innervated by the sympathetic nervous system (SNS)^7,10^. HRV is the fluctuation in the length of the heartbeat R-R intervals, reflecting the involvement of ANS activity in cardiac regulation^8,9^. Thus, PLR mainly reflects the central autonomic regulation, while HRV reflects the peripheral autonomic regulation. Tests assessing these parameters might be effective in exploring the association between CRS and the central/peripheral ANS activity.

Deciding the study design and methods can be one of the critical issues in exploring the association between PLR/HRV parameters and CRS. SNOT-22 questionnaire scores represent CRS-specific quality of life (QOL)^19^ and have been used as the primary outcome in prospective^20,21^ and longitudinal^22^ clinical studies on CRS. As treatments including surgical intervention can result in long-term improvements in CRS-specific QOL^22^, there might be close associations between CRS-specific QOL and ANS activity. Therefore, a prospective longitudinal study investigating the association between SNOT-22 scores and PLR/HRV parameters in patients with CRS undergoing treatments, including surgical intervention, can contribute to exploring the relation between ANS activity and CRS. In a longitudinal study, data can be collected within a predefined period. Therefore, the data can show information about the degree of variations across time for each patient (within-individual variability) and among patients (between-individual variability). The use of linear mixed-effects models (LMMs) can be an appropriate statistical method for repeated data because LMMs possess the flexibility for assumptions and the advantage of adjusting individual variabilities^23,24^. Especially, as adjusting the individual variability can reduce false positives and false negatives in the obtained data^24^, the use of LMMs in a longitudinal study or repeated data can lead to robust conclusions without underestimating or overestimating the results.

The current study aimed to establish objective indicators of CRS status with the use of SNOT-22 and PLR/HRV parameters. For this purpose, we designed a 12-month prospective longitudinal study targeting patients with CRS who underwent treatments including surgical intervention and investigated the effects of adjusting the individual variability in results using two different statistical models.

## Materials and methods

### Patients

The Research Ethics Committee of Hyogo Prefectural Amagasaki General Medical Center approved this prospective longitudinal study.

Patients who underwent surgical intervention for medically refractory CRS at Hyogo Prefectural Amagasaki General Medical Center between August 2018 and April 2021 were enrolled. We performed this study in accordance with the tenets of the Declaration of Helsinki.

We collected sociodemographic and medical characteristics data through questionnaires, including sex, age, body mass index (BMI), smoking intensity according to the Brinkman index (number of cigarettes smoked per day multiplied by the number of smoking years), asthma, and diabetes. All patients signed an informed consent form after receiving information about the purpose and procedures of the study.

The inclusion criteria were age > 18 years and CRS that had been resistant to medical treatments for > 3 months. All patients underwent a biopsy when nasal polyps were observed endoscopically. The Japanese Epidemiological Survey of Refractory Eosinophilic Chronic Rhinosinusitis scoring system was preoperatively applied to the clinical data and biopsy samples to determine whether the patients had eCRS or neCRS^25^. The exclusion criteria were histories of glaucoma, arrhythmia, and cardiovascular disorders requiring a pacemaker. When enrolled patients did not visit postoperatively, these patients were excluded from the analysis.

BMI was classified into three categories^26^: (i) underweight, BMI < 18.5 kg/m^2^; (ii) healthy weight, 18.5 ≤ BMI < 25.0 kg/m^2^; and (iii) overweight and obese, 25 ≤ BMI kg/m^2^. Asthma status was classified as follows: (i) no asthma or (ii) inactive or active asthma. An inactive status was defined as having a history of asthma or asthma in remission, whereas an active status was defined as having current asthma requiring follow-up and/or treatments with specialists under the Japanese guidelines for adult asthma^27^. Diabetes status was classified as follows: (i) without diabetes or (ii) with diabetes requiring education for lifestyle changes, medication including oral hypoglycaemic agents, and insulin. CRS was divided into two phenotypes: (i) eCRS and (ii) neCRS.

### Subjective and objective tools

All patients underwent subjective and objective measurements. As a subjective measurement tool, we used SNOT-22, which is a validated questionnaire that includes the 22 symptoms most related to CRS-specific QOL^19^. All symptoms were scored from 0 to 5 (0 = ‘no problem’, 1 = ‘very mild problem’, 2 = ‘mild or slight problem’, 3 = ‘moderate’, 4 = ‘severe problem’, and 5 = ‘problem as bad as it can be’). Higher scores represent worse CRS-specific QOL, and lower scores indicate improved QOL.

A hand-held infrared pupillometer (PLR-3000; NeurOptics, Irvine, CA, USA) and an HRV device (CheckMyHeart; Daily Care BioMedical, Taiwan) were used as objective measurement tools for the PLR and HRV tests, respectively. The details of the systems and tests have been previously described^17,18^.

In the PLR test, we assessed the following six parameters (Fig. 3A): (i) INIT (mm); (ii) ACV (mm/s); (iii) MCV (mm/s); (iv) constriction ratio, defined as the difference between the INIT and the minimum pupil size divided by the INIT (DELTA, %); (v) LAT (s); and (vi) ADV (mm/s). As PLR parameters can be affected by different light stimulus intensities (Fig. 3B)^17,18^, we used four different stimulus intensities using a flashlight with a fixed 800-ms pulse duration: (i) 10 μW, (ii) 50 μW, (iii) 121 μW, and (iv) 180 μW. The recording duration was 5 s. The following PLR parameters might be used as indicators of ANS activity: INIT as an indicator of sympathovagal balance^28^ and MCV^10^, DELTA^10^, and LAT^29^ as indicators of PNS activity.

In the HRV test, data recorded by the HRV device for 5 min (sampling rate: 250/s) were collected and analyzed using the software provided by the manufacturer (CheckMyHeart, Daily Care BioMedical). The software rejected the irregular R–R intervals automatically. The R–R intervals (resolution 4 ms) were obtained in the software. Cubical interpolation and resampling at 1 Hz were performed for the detrended time series. HRV analysis involves two different methods for assessing ANS activity: (i) time-domain analysis and (ii) frequency domain analysis. After the detrending procedures, the power spectral density of R-R intervals was calculated by an autoregressive method and a fast Fourier transform method. The order of autoregressive method was set as 16. We used three detrend parameters in the time-domain analysis: (i) standard deviation of the normal normal-to-normal (NN) intervals (SDNN, ms), (ii) root mean square of the successive NN interval differences (rMSSD, ms), and (iii) pNN50. We obtained five detrend parameters quantified using the autoregressive method for the frequency domain analysis: (i) low-frequency power (LF power, 0.04–0.15 Hz); (ii) high-frequency power (HF power, 0.15–0.4 Hz); (iii) normalized values of LF, (LF norm; LF/total power − very low frequency) × 100; (iv) normalized values of HF, (HF norm; HF/total power − very low frequency) × 100; and (v) LF/HF values. Previously, raw values such as LF and HF powers have been reported to have disadvantages due to large inter-variability and intra-variability, resulting in a long-tailed right-skewed exponential distribution^8,17^. Therefore, we excluded LF and HF powers. Finally, we adopted six HRV parameters for analysis: (i) SDNN, (ii) rMSSD, (iii) pNN50, (iv) LF norm, (v) HF norm, and (vi) LF/HF. The following parameters can be used as indicators of ANS activity: rMSSD, pNN50, and HF norm as indicators of PNS activity; SDNN and LF norm as an indicator of SNS and PNS activity modulation; and LF/HF as an indicator of sympathovagal balance^8,9^. However, these concepts have been recently criticized because of the non-linear interactions between SNS and PNS activity^30^. Thus, the interpretation of the HF norm, LF norm, and LF/HF as autonomic indicators remains debatable.

### Treatments for CRS

The surgical strategies were determined on the basis of computed tomographic and endoscopic findings. All patients underwent endoscopic sinus surgery (ESS). The procedures consisted of either unilateral or bilateral maxillary antrostomy, ethmoidectomy, frontal sinusotomy, or sphenoidotomy, with septoplasty and inferior turbinate surgery as adjunctive procedures when needed. The patients underwent ESS under general anesthesia and received nasal packing (Merocel; Medtronic Xomed, Minneapolis, MN, USA) on the surgical side just before awakening to prevent postoperative bleeding. The nasal packing was removed 1 day after ESS. As a postoperative care procedure, all patients were recommended to perform saline irrigation during the follow-up period. Some patients, especially those with eCRS, can experience recurrence even after ESS^31^. When patients required additional treatments because of postoperative recurrence, a short course of systemic corticosteroid therapy (oral betamethasone: 1 mg/day for 1 week) or antibiotic therapy was administered. The number of the systemic corticosteroid medication courses was limited to two per month.

### Study design

The PLR and HRV tests were performed in a seated position in a quiet consulting room kept at a comfortable temperature (21–25 °C). The patients were required to keep their position in a relaxed state during the tests. All patients received the short-term HRV test, followed by the PLR test.

We performed PLR and HRV tests at eight time points: (i) 1 week before ESS (defined as a reference), (ii) 1 week after ESS, (iii) 2 weeks after ESS, (iv) 1 month after ESS, (v) 2 months after ESS, (vi) 3 months after ESS, (vii) 6 months after ESS, and (viii) 12 months after ESS. The assessment time was between 8 am and 12 pm at all time points to minimize the effects of the circadian rhythm.

### Statistical analysis

We expressed quantitative variables as means and standard deviations/95% CIs or as medians and interquartile ranges. We compared the SNOT-22 total symptom scores as non-parametric data across the time points using the Friedman test with Dunn’s multiple comparison test.

We investigated the association between SNOT-22 scores and PLR/HRV parameters using linear regression models. To reveal the effects of adjusting individual variability, we prepared two models: (i) linear regression model adjusting clinical factors as predictor variables (model 1) and (ii) LMM adjusting clinical factors and individual variability (model 2). The linear regression model consisted of fixed effects as predictor variables, whereas the LMM consisted of fixed and random effects. Random effects were subdivided into random intercepts and slopes, although the random slope models can be more prone to convergence problems than the random intercept models^24^. Therefore, we selected the random intercept model for the LMM. We introduced the total SNOT-22 scores, sex, age, smoking intensity, BMI categories, CRS phenotypes, measured eye side (only for PLR analyses), light stimulus intensity (only for PLR analyses), and the status of asthma and diabetes as fixed effects, whereas each patient ID was used as random effects for adjusting individual variability. PLR/HRV parameters were applied as response variables. The SNOT-22 total symptom scores, values of PLR and HRV parameters, light stimulus intensity, age, and smoking intensity were used as continuous variables, whereas sex, measured eye side, CRS phenotypes, and status of asthma and diabetes were used as binary variables. BMI was used as a categorical variable. To evaluate the effects of adjusting among-individual variability, we compared AIC values and regression coefficients of PLR/HRV parameters for every 1 unit increase in the SNOT-22 total symptom score. The model with the lower AIC values was defined as the better-fit model^32^. A two-sided *p*-value of < 0.05 was considered statistically significant. All statistical analyses were performed with R (version 3.6.1; R Foundation for Statistical Computing, Vienna, Austria; lme4 and lmerTest packages).

### Ethics approval and consent to participate

The Research Ethics Committee of Hyogo Prefectural Amagasaki General Medical Center approved this study (approval number: 2-184). We obtained written informed consents from all subjects.

## Results

### Characteristics of patients

A total of 67 Japanese patients were enrolled in the current study. Table 1 shows the sociodemographic and medical characteristics of the patients.

**Table 1:** Sociodemographic and medical characteristics of the patients.

Characteristics		Patients (N = 67)
Median age (IQR)		58 (46–70)
Sex, male, n (%)		40 (60)
**Body mass index, n (%)**		
Underweight: < 18.5 kg/m^2^		5 (7)
Healthy: 18.5 kg/m^2^ ≤ body mass index (BMI) < 25.0 kg/m^2^		36 (54)
Overweight and obese: BMI ≥ 25.0 kg/m^2^		26 (39)
Smoking intensity, Brinkman index, median (IQR)		120 (0–430)
**Asthma status, n (%)**		
No asthma		47 (70)
Inactive or active asthma		20 (30)
**Diabetes status, n (%)**		
Without diabetes		59 (88)
With diabetes		8 (12)
**Chronic rhinosinusitis phenotype**		
Non-eosinophilic		42 (63)
Eosinophilic		25 (37)

*IQR* interquartile range.

### Changes in total SNOT-22 scores over time

**Figure 1:**
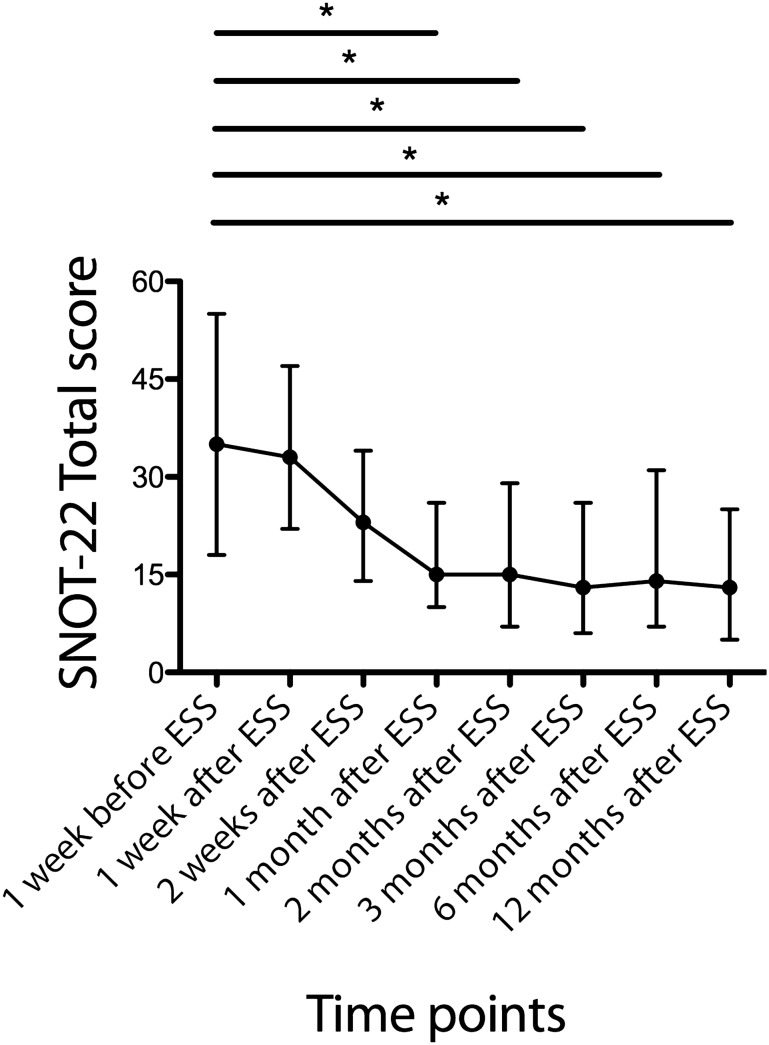
Changes in SNOT-22 total symptom scores over time. Data are presented as medians and interquartile ranges. Results of post-hoc analysis with Dunn’s multiple comparison test: *p < 0.001. *ESS* endoscopic sinus surgery, *SNOT-22* 22-item Sino-Nasal Outcome Test.

We compared the SNOT-22 total symptom scores across the eight time points (Fig. 1). The scores significantly changed during the follow-up period (*p* < 0.001). Compared with the preoperative values, post-hoc tests revealed a significant decrease in the scores from postoperative 1 to 12 months, indicating an improvement in CRS-specific QOL after treatments.

### Association between SNOT-22 scores and PLR/HRV parameters

To establish PLR/HRV parameters as objective indicators reflecting CRS status, we investigated the association between SNOT-22 total scores and PLR/HRV parameters using two linear regression models. In the comparison of AIC values between the two models, model 2 showed lower Akaike’s information criterion (AIC) values for all parameters (Tables 2 and 3). Therefore, we defined model 2 as the better-fit model.

**Table 2:** Comparison of Akaike’s information criterion and regression coefficients between the two models for pupillary light reflex parameters.

Parameter (unit)		INIT (mm/score)	ACV (mm/s/score)	MCV (mm/s/score)
AIC	Model 1	8720	5544	9268
	Model 2	2932	2726	6878
Regression coefficient (95% CI)	Model 1	2.0*** (0.9, 3.2) × 10^–3^	1.8*** (1.0, 2.6) × 10^–3^	3.6*** (2.3, 4.8) × 10^–3^
	Model 2	− 0.2 (− 0.9, 0.5) × 10^–3^	1.2*** (0.6, 1.9) × 10^–3^	3.5*** (2.4, 4.6) × 10^–3^

Parameter (unit)		DELTA (%/score)	LAT (s/score)	ADV (mm/s/score)
AIC	Model 1	25,938	− 19,669	− 231
	Model 2	23,740	− 20,377	− 2216
Regression coefficient (95% CI)	Model 1	− 9.0* (− 17.6, − 0.4) × 10^–3^	− 1.4*** (− 1.8, − 1.0) × 10^–4^	1.1*** (0.7, 1.5) × 10^–3^
	Model 2	14.4*** (6.2, 22.4) × 10^–3^	− 1.8*** (− 2.2, − 1.3) × 10^–4^	0.9*** (0.6, 1.3) × 10^–3^

*ACV* average constriction velocity, *ADV* average dilation velocity, *AIC* Akaike’s information criterion, *CI* confidence interval, *DELTA* constriction ratio, *INIT* initial pupil size, *LAT* constriction latency, *MCV* maximum constriction velocity.

*p < 0.05; **p < 0.01; ***p < 0.001.

**Table 3:** Comparison of Akaike’s information criterion and regression coefficients between the two models for heart rate variability parameters.

Parameter (unit)		SDNN (ms/score)	rMSSD (ms/score)	pNN50 (%/score)
AIC	Model 1	4853	4780	3953
	Model 2	4594	4672	3631
Regression coefficient (95% CI)	Model 1	10.1 (− 0.8, 20.9) × 10^–2^	7.4 (− 2.8, 17.5) × 10^–2^	10.5*** (5.9, 15.2) × 10^–2^
	Model 2	− 4.3 (− 13.3, 5.3) × 10^–2^	− 1.2 (− 10.9, 9.4) × 10^–2^	2.3 (− 1.3, 6.1) × 10^–2^

Parameter (unit)		LF norm (1/score)	HF norm (1/score)	LF/HF (1/score)
AIC	Model 1	4509	4508	2243
	Model 2	4431	4428	2219
Regression coefficient (95% CI)	Model 1	− 3.9 (− 11.7, 4.0) × 10^–2^	3.7 (− 4.2, 11.6) × 10^–2^	− 4.4 (− 14.0, 5.1) × 10^–3^
	Model 2	− 3.5 (− 11.5, 4.5) × 10^–2^	3.1 (− 4.9, 11.1) × 10^–2^	− 3.9 (− 13.7, 5.7) × 10^–3^

*AIC* Akaike’s information criterion, *CI* confidence interval, *HF norm* normalised values of high-frequency heart rate variability, *LF norm* normalised values of low-frequency, numeric rating scale, *pNN50* percentage of adjacent N–N intervals that differ from each other by > 50 ms, *rMSSD* root mean square of successive normal-to-normal interval differences, *SDNN* standard deviation of normal normal-to-normal intervals.

*p < 0.05; **p < 0.01; ***p < 0.001.

**Figure 2:**
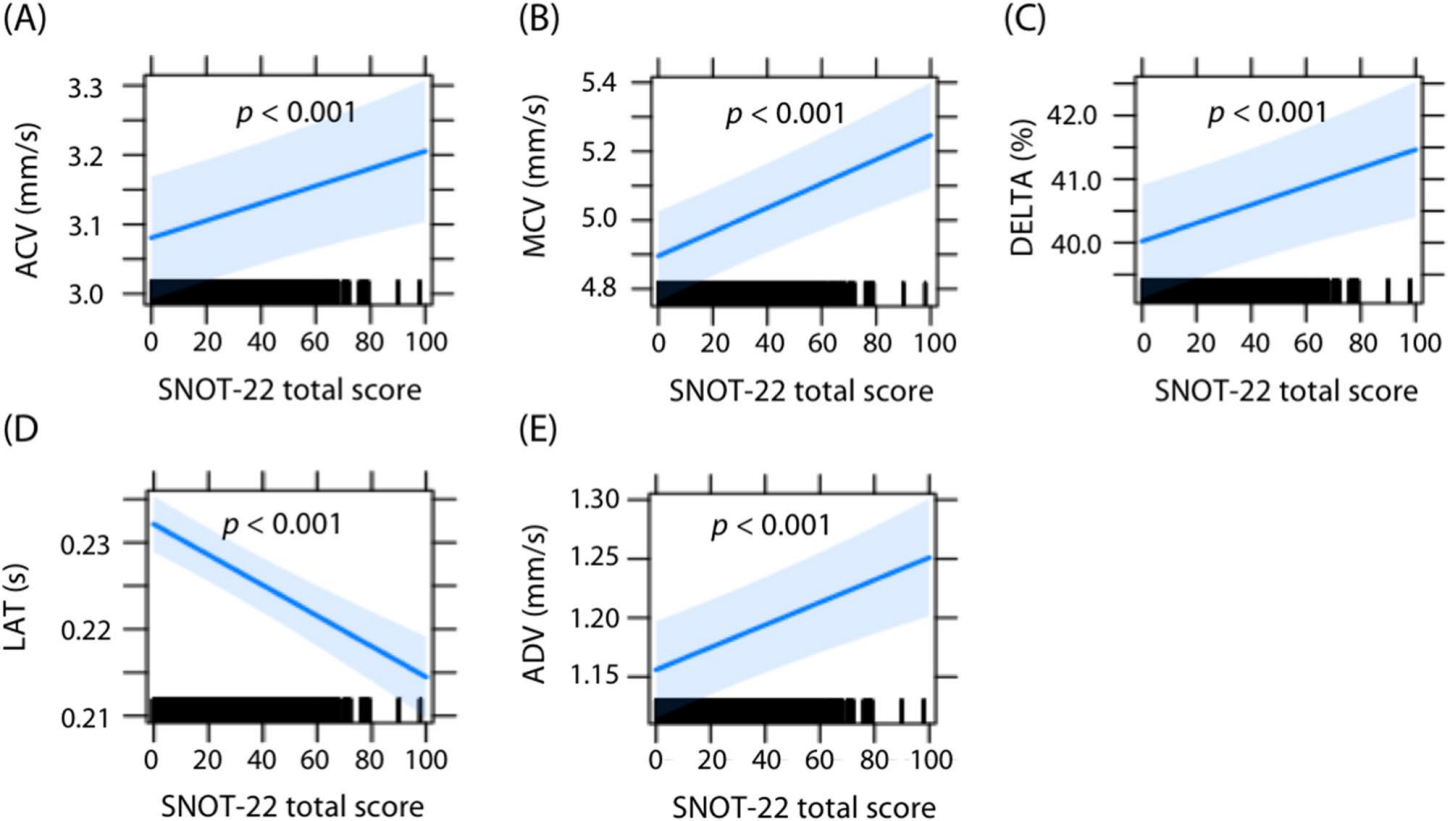
Association between SNOT-22 total symptom scores and pupillary light reflex parameters showing significant regression coefficients. The graph shows parameter slopes for changes in SNOT-22 total symptom scores with 95% confidence intervals. (**A**) ACV, (**B**) MCV, (**C**) DELTA, (**D**) LAT, and (**E**) ADV. *ACV* average constriction velocity, *ADV* average dilation velocity, *DELTA* constriction ratio, *LAT* constriction latency, *MCV* maximum constriction velocity, *SNOT-22* 22-item Sino-Nasal Outcome Test.

To evaluate the effects of adjusting individual variability with LMMs, we compared the regression coefficients of PLR/HRV parameters for every 1 unit change in the SNOT-22 total symptom scores (Tables 2 and 3). In PLR/HRV analyses, both models showed significances in five PLR parameters (see Models 2 in Table 2) but not in any HRV parameter (see Models 2 in Table 3). Initial pupil size (INIT) in PLR and percentage of adjacent normal-to-normal intervals that differ from each other by > 50 ms (pNN50) showed opposite results between the two models: model 1 showed significances but not model 2. In the constriction ratio (DELTA), model 1 showed a significance with negative values, while model 2 showed one with positive values. As shown in Fig. 2, decreased SNOT-22 total scores were associated with significant decreases in average constriction velocity (ACV), maximum constriction velocity (MCV), DELTA, and average dilation velocity (ADV), and prolonged constriction latency (LAT). Out of these PLR parameters, LAT showed the narrowest 95% confidence intervals (CIs).

## Discussion

We assessed the association between PLR/HRV parameters and SNOT-22 total symptom scores using two statistical models in this prospective longitudinal study targeting patients with CRS. The results showed that model 2 had lower AIC values in all parameters. Three parameters showed opposite results between the two models: DELTA, INIT, and pNN50. Moreover, model 2 showed significant parameter changes with altered SNOT-22 scores in five PLR parameters but not HRV parameters. Among the PLR parameters, LAT showed the narrowest 95% CIs.

This study showed that the better-fit model of model 2 for all parameters and the opposite results between the two models in three parameters. These findings indicated that adjusting the among-individual variability with LMMs can improve the model fit, contributing to the reduction of false-positive ratios and the prevention of misleading conclusions. The findings were consistent with our previous study^33^.

Previously, we investigated whether PLR/HRV parameters can be altered by conditions that create a physiological burden, such as pain^18^ and foot bath^17^. Both PLR and HRV parameters showed significant alterations with altered subjective pain intensity scores in our previous study targeting clinical pain relief models^18^. However, our previous study investigating the effects of foot bath demonstrated significant alterations after foot bath in five out of seven PLR parameters but in only a few HRV parameters^17^. These findings indicated the importance of evaluating ANS activity targeting several organs and tissues. Our current study showed significant regression coefficients with altered SNOT-22 scores in five PLR parameters but not in any HRV parameters. The different responses might be due to the different sensitivities to altered CRS-specific QOL between PLR and HRV parameters. PLR parameters might be more effective than HRV parameters in assessing the association between CRS-specific QOL and ANS activity. Among the five PLR parameters, the narrowest 95% CIs was observed in LAT (see Fig. 2). The findings indicated that LAT is the most robust indicator reflecting the CRS-specific QOL.

**Figure 3:**
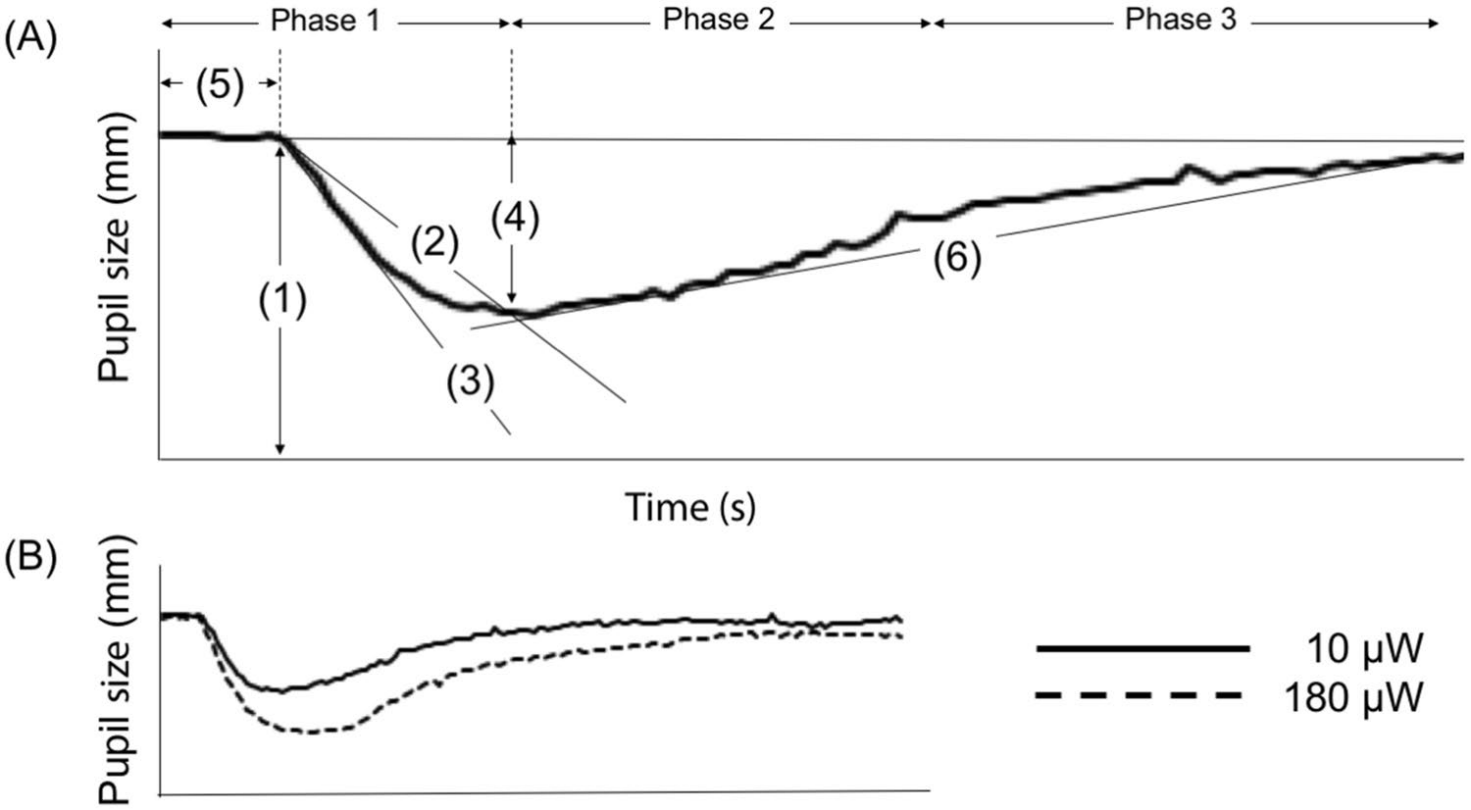
Pupillary light reflex wave. (**A**) (1) initial pupil size (INIT); (2) average constriction velocity (ACV); (3) maximum constriction velocity (MCV); (4) constriction ratio, defined as the difference between the initial and minimum pupil sizes divided by the initial pupil size (DELTA); (5) constriction latency (LAT); and (6) average dilation velocity (ADV). The wave consists of three phases. (**B**) Waves of pupillary light reflex recorded at 10 and 180 μW. Solid line represents a wave recorded at 10 μW, whereas dotted line represents a wave recorded at 180 μW.

Researchers may be interested in interpreting parameter alterations from the viewpoint of ANS activity. Although some HRV parameters remain debatable as autonomic roles (HF norm, LF norm, and LF/HF)^30,34^, this study showed that the altered ANS activity in the heart due to treatments can be less intense than that in the pupil. In PLR wave, the three phases are under the control of PNS and SNS activities (Fig. 3A): (i) phase 1 is mainly controlled by PNS activity; (ii) phase 2 is controlled by PNS and SNS activities; and (iii) phase 3 is predominantly controlled by SNS activity^10^. Therefore, parameters obtained from phase 1, such as ACV, MCV, DELTA, and LAT, can be used as indicators of PNS activity. For these parameters, a literature review reported MCV and relative constriction amplitude (i.e., DELTA) as indicators sensitive to PNS activity but questioned whether the same is true for LAT^10^. Previously, we investigated how PLR parameters can be altered by different light stimulus intensities in healthy individuals^17^. We observed shortened LAT and increased ACV, MCV, and DELTA with increased light stimulus intensity. As pupillary constriction can be a consequence of increased PNS outputs^10^, changes in LAT, ACV, MCV, and DELTA can be considered to indicate increased PNS activity^17^. As ADV is a parameter obtained from phase 2/3, it can be affected by SNS and PNS activities. However, the role of ADV as an indicator of ANS activity remains unclear. Taking these findings together, five PLR parameters can effectively reveal the association between CRS and ANS activity: ACV, MCV, DELTA, LAT, and ADV. The interpretation of altered PLR parameters from viewpoints of ANS activity requires attention as some PLR parameters remain unclear as autonomic indicators with reliability as well as HRV parameters. Prolonged LAT and decreased ACV, MCV, and DELTA due to decreased SNOT-22 scores, as observed in the current study, might be considered to indicate decreased PNS activity due to improvement of CRS-specific QOL.

This study had several limitations. First, the PLR alterations observed in the current study may have been affected by unconsidered factors such as psychological status^35^. As the five symptom domains, including psychological dysfunction, were defined using SNOT-22^36^, assessing the association between the PLR parameters and the domain might be effective in revealing the effects of psychological status on PLR parameters. In addition, the measurement was performed at a single time point before treatment. Preoperative measurements at several time points should be performed, and test–retest variability should be assessed. Second, this was designed as a one-group pre-treatment versus post-treatment longitudinal study. Therefore, it remains unclear whether the PLR parameter alterations observed in our current study can represent hyperactivity of the PNS in patients with CRS because we targeted a single CRS group. Further studies including several groups might overcome these issues. Third, we used SNOT-22 to evaluate the effects of CRS treatment. Although SNOT-22 can be an effective subjective tool for analyzing the CRS status, the scores represent CRS-specific QOL but not ANS dysfunction. Therefore, the use of ANS-focused questionnaires, such as the COMPASS-31, might provide different insights into the association between CRS-related PLR/HRV parameter alterations and ANS activity. Fourth, the current study data were obtained from a small sample size. Since eosinophilic CRS (eCRS) has close associations with type 2 immune responses^31^, and type 2 immune responses can be modulated by acetylcholine derived from PNS activity^37^, researchers might be interested in the differences in PLR/HRV parameter values between non-eCRS (neCRS) and eCRS. Future studies with larger samples for both neCRS and eCRS would enable assessing whether the CRS phenotype can affect ANS activity. Fifth, it was impossible to discuss the associations between SNS activity and CRS because PLR is predominantly controlled by PNS activity^10^. As INIT is an indicator of sympathovagal balance^28^, the lack of significant alterations in INIT observed in this study might reveal the decreased SNS and PNS activities with the same extent (non-linear interactions between SNS and PNS activity).

In summary, this study revealed the clinical potential of five PLR parameters as objective indicators of CRS-specific QOL, with LAT being the most robust. To obtain robust conclusions from PLR/HRV data obtained from a longitudinal study, not only clinical factors but also individual variability should be adjusted. As the autonomic roles in some parameters remain unclear, further studies are necessary to reveal the association between CRS and ANS activity.

## Data Availability

The datasets are available from the corresponding author upon request.

## Abbreviations

ACV: Average constriction velocity
ADV: Average dilation velocity
AIC: Akaike’s information criterion
ANS: Autonomic nervous system
BMI: Body mass index
COMPASS-31: 31-Item Composite Autonomic Symptom Score
CRS: Chronic rhinosinusitis
DELTA: Constriction ratio
eCRS: Eosinophilic chronic rhinosinusitis
ESS: Endoscopic sinus surgery
HF: High frequency
HRV: Heart rate variability
INIT: Initial pupil size
LAT: Constriction latency
LF: Low frequency
LMM: Linear mixed-effects model
MCV: Maximum constriction velocity
neCRS: Non-eosinophilic chronic rhinosinusitis
norm: Normalised
PLR: Pupillary light reflex
pNN50: Percentage of adjacent normal-to-normal intervals that differ from each other by > 50 ms
PNS: Parasympathetic nervous system
QOL: Quality of life
rMSSD: Root mean square of successive normal-to-normal interval differences
SDNN: Standard deviation of normal normal-to-normal intervals
SNOT-22: 22-Item Sino-Nasal Outcome Test
SNS: Sympathetic nervous system
